# Antithrombotic strategies after transcatheter aortic valve replacement a network meta-analysis

**DOI:** 10.3389/fcvm.2025.1496334

**Published:** 2025-05-09

**Authors:** Mengxiao Shi, Ying Wu, Qing Zhou

**Affiliations:** ^1^ Department of Cardio-Thoracic Surgery, Nanjing Drum Tower Hospital Clinical College of Nanjing University of Chinese Medicine, Nanjing, Jiangsu, China; ^2^ Department of Cardio-thoracic Surgery, Nanjing Drum Tower Hospital, Nanjing, China

**Keywords:** TAVR, antithrombotic therapy, antiplatelet therapy, anticoagulation therapy, meta-analysis

## Abstract

The optimal antithrombotic regimen for patients without an indication for oral anticoagulation (OAC) after transcatheter aortic valve replacement (TAVR) remains unclear. We conducted a network meta-analysis of randomized controlled trials to clarify the best postoperative antithrombotic regimen. We searched literature databases including PubMed/Medline and Cochrane up to June 2024. Safety endpoints included all-cause death, cardiovascular death, major/life-threatening bleeding, and minor bleeding during follow-up. Efficacy endpoints encompassed ischaemic stroke transient ischemic attack (TIA), systemic embolism, hypoattenuated leaflet thickening (HALT), and reduced leaflet motion (RELM). Outcomes were assessed during the follow-up period specified in each trial (range:3–24 months). The results were statistically analyzed using R 4.3.2 and Stata 16 software. The final analysis included seven randomized controlled trials. Single antiplatelet therapy (SAPT) showed a lower incidence of major/life-threatening bleeding compared to the direct oral anticoagulants (DOACs) group (OR: 0.68, 95% CI: 0.47–0.99, *P* = 0.002) during the 3–24 month post-TAVR period. However, no significant differences were found in other safety or efficacy endpoints. SAPT is the preferred treatment strategy for TAVR patients without anticoagulation indications.

**Systematic Review Registration:**

identifier, PROSPERO registration number: CRD42024584735.

## Introduction

TAVR has become the leading transcatheter approach for replacing a narrowed aortic valve in patients with severe symptomatic aortic stenosis (1, 2). The success rate of the TAVR procedure has improved over time; however, it remains associated with long-term cardiovascular complications requiring targeted antithrombotic management, such as myocardial infarction, stroke, major bleeding, valve thrombosis, and systemic embolism (3, 4).

Optimal medical management of patients after TAVR is crucial, focusing on antithrombotic therapy to prevent valve thrombosis and other complications while balancing bleeding risks. Guidelines from the American College of Cardiology (ACC) and the American Heart Association (AHA) in 2020 recommended SAPT after TAVR for patients without an indication for OAC (5). Recent randomized controlled trials have shown that SAPT after TAVR in patients without an indication for OAC did not increase the risk of ischemic events and reduced bleeding risk compared to dual antiplatelet therapy (DAPT) (1).

Recent studies indicate that DOAC reduce the incidence of HALT and RELM, which are imaging biomarkers associated with subclinical valve thrombosis. However, current randomized controlled trials (RCTs) have not demonstrated conclusive evidence that DOACs improve long-term valve durability or hemodynamic performance. For instance, the GALILEO trial showed a reduction in HALT and RELM with rivaroxaban compared to antiplatelet therapy, but no significant difference in clinical valve dysfunction endpoints was observed (6). A limitation of existing studies is the heterogeneity of control groups, where combinations of DAPT, SAPT, and VKA may introduce confounding factors. This heterogeneity complicates the direct comparison of antithrombotic strategies and their impact on valve outcomes (7).

A meta-analysis suggested that SAPT is preferable after TAVR, although it was compared with the DAPT group, and did not address important thrombotic events like HALT and RELM (8). Few randomized trials have tested DOACs for preventing thromboembolic events after TAVR in recent years (9).

Therefore, it remains unclear whether SAPT, DAPT, DOACs, or SAPT combined with VKA are superior, particularly regarding the balance between bleeding and thrombotic events. To clarify this equipoise, we conducted a meta-analysis comparing the risks and benefits of various antithrombotic regimens after TAVR, particularly compared to DOACs. This analysis focused on recent randomized controlled trials involving patients without indications for anticoagulation.

## Methods

### Study design

This systematic review and meta-analysis was performed in adherence to the Preferred Reporting Items for Systematic Reviews and Meta-Analyses (PRISMA) guidelines (10) and the Cochrane Handbook for Systematic Reviews of Interventions (11). Given the nature of this study as a secondary analysis of published data, formal ethical approval and patient consent were waived.

### Database search

This systematic review was registered with PROSPERO in August 2024 (PROSPERO registration number: CRD42024584735). We searched PubMed/Medline and the Cochrane Library for randomized controlled trials comparing antithrombotic strategies after TAVR up to 1 June 2024. Detailed search strategies are provided in Supplementary Table S1.

### Risk of bias assessment

The Cochrane Risk of Bias-2 (ROB-2) tool (10) was used to evaluate bias across all included trials. Studies were appraised based on six predefined domains: (1) random sequence generation, (2) allocation concealment, (3) participant blinding, (4) personnel blinding, (5) selective reporting, and (6) incomplete outcome data. Each domain was classified as having low, high, or unclear risk of bias, with an overall risk-of-bias summary provided for each study. Two independent reviewers performed the bias assessment, with discrepancies resolved through consensus discussion.

### Inclusion and exclusion criteria

Inclusion criteria: (1) post-TAVR patients without indications for anticoagulation. (2) type of study was a randomized controlled study. (3) groups receiving various antithrombotic therapies. (4) Reported outcomes encompassed safety endpoints: all-cause death, cardiovascular death, major/life-threatening hemorrhage, minor hemorrhage, and efficacy over follow-up. Additional endpoints were ischemic stroke/TIA, systemic embolism, HALT, and RELM events.

Exclusion criteria: (1) patients needing OAC anticoagulation. (2) non-randomized controlled studies (e.g., retrospective analyses). (3) any conditions deemed unsuitable by investigators.

DOAC served as the control group, with other antithrombotic strategies compared against it.

### Research screening

All identified citations were imported into EndNote for deduplication. Two researchers independently reviewed the titles and abstracts to ensure compliance. Discrepancies in the study selection process were resolved through consultation and consensus with the other authors.

### Data extraction

Data extraction was obtained in the included RCTs. This included extracting trial characteristics (authors, publication year, study design, subject numbers, experimental and control groups, follow-up duration), study baseline characteristics [age, sex, BMI, comorbidities, Society for Thoracic Surgery (STS) score, European Cardiovascular Surgery Risk Factor Score EURO SCORE], and the predefined endpoints. The authors employed a standardized extraction form, resolving discrepancies through consensus.

### Statistical analysis

Statistical analyses were performed using R version 4.3.2 and Stata 16. Dichotomous outcomes were pooled using the odds ratio (OR) and 95% confidence interval (CI) as effect measures. A random-effects model was applied for meta-analyses. Statistical significance was defined as *p* < 0.05.

## Result

### Search results incorporated

**Figure 1:**
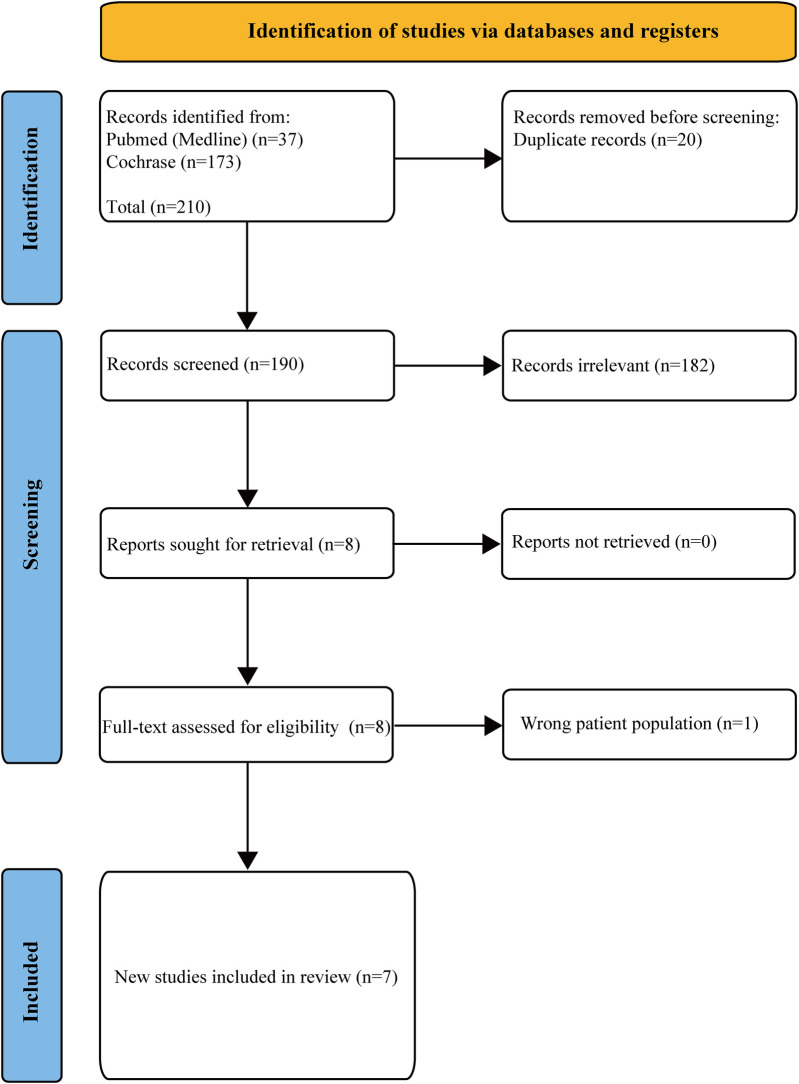
PRISMA flow chart in the screening process.

A total of 210 studies were retrieved from PubMed/Medline (37) and Cochrane (173). Twenty duplicate studies were removed; One hundred and eighty-two irrelevant articles were excluded based on non-RCT designs, non-TAVR populations, or lack of antithrombotic strategy comparisons; there were no unretrieved reports. Among the 8 studies, one study with an incorrect population was excluded, and finally 7 studies were included. The detailed PRISMA flowchart process is depicted in Figure 1.

**Figure 2:**
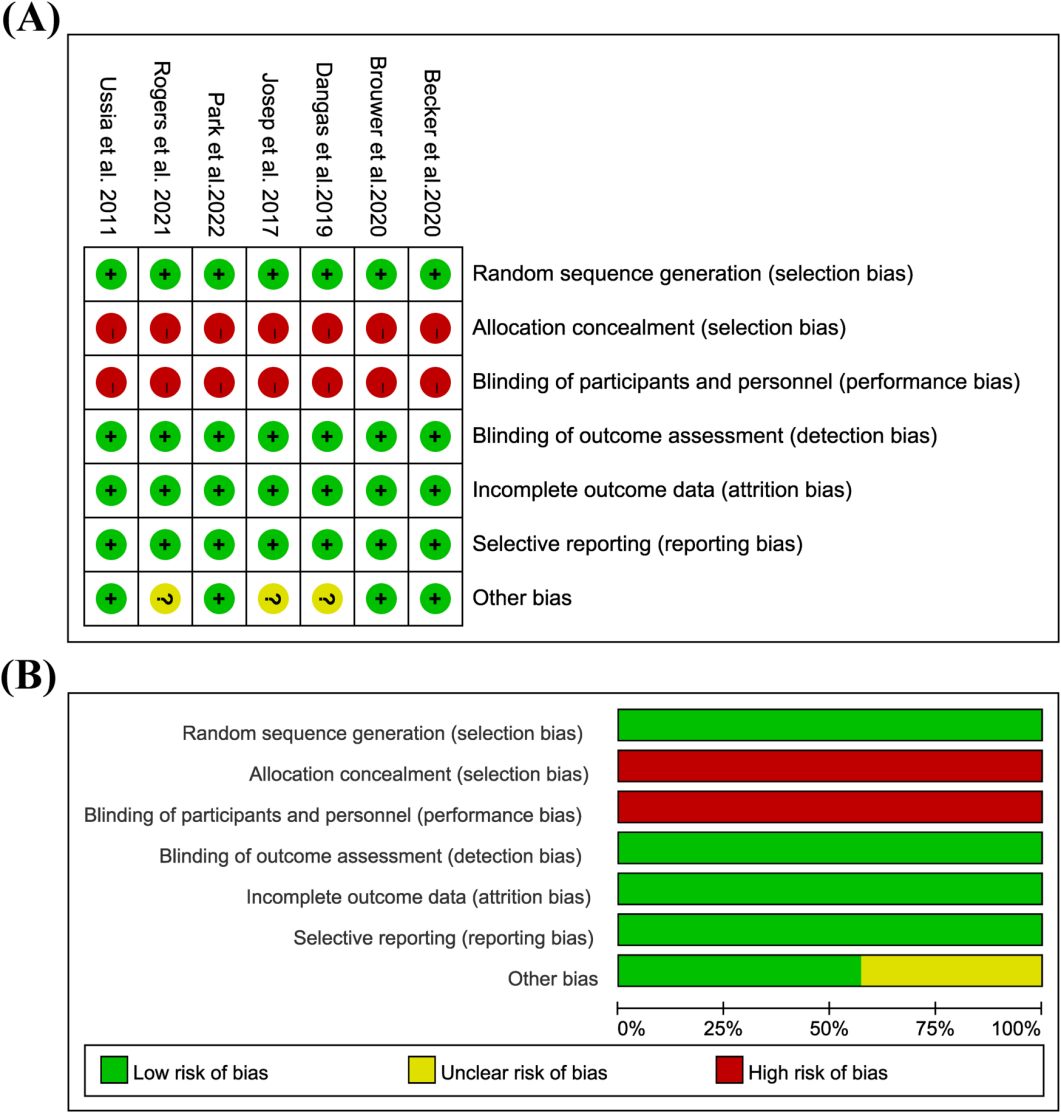
Bias risk assessment in the study: **(A)** risk of bias graph; **(B)** risk of bias summary; low = green; unclear = yellow; height = red.

### Characteristics of inclusion in the study

Seven randomized controlled trials involving 3,164 patients were included (6, 12–17). 602 cases in the DAPT group, 1,052 in the DOAC group, 1,463 in the SAPT group, and 44 in the VKA + SAPT group. The characteristics of the included research are shown in Table 1. The baselines included in the study are shown in Table 2.

**Table 1:** Incorporate research characteristics.

Study ID	Total patients	Experimental group	Drug dose	Control group	Drug dose	Follow up (Months)	Primary outcome	TAVI access
Duk-Woo Park et al. 2022 (12) (ADAPT-TAVR）	229	DAPT	Aspirin (100 mg) plus clopidogrel (75 mg) Once daily	Edoxaban	Edoxaban 60 or 30 mg Once daily	6	Valve leaflet thrombosis	Transfemoral
George D. Dangas et al. 2019 (6) (GALILEO)	1644	SAPT	Aspirin at a dose of 75–100 mg daily and clopidogrel at a dose of 75 mg. once daily for 3 months. Followed by aspirin monotherapy (75–100 mg daily)	Rivaroxaban	Rivaroxaban at a dose of 10 mg daily plus aspirin at a dose of 75–100 mg daily for 3 months, followed by rivaroxaban monotherapy (10 mg daily)	24	The composite of all-cause mortality, MI, stroke/TIA, SE, valve thrombosis, and DVT/PE	N/A
Gian Paolo Ussia et al. 2011 (13) USSIA）	79	SAPT	Aspirin 100 mg Once daily	DAPT	75 mg clopidogrel plus aspirin 100 mg	6	The primary the endpoint was the composite of major adverse cardiac and cerebrovascular events (MACCE)	There were 77 patients in the transfemoral group. Among them, there were 38 patients in the DAPT group and 39 patients in the SAPT group. There were 2 patients in the trans- subclavian group, and both of them were in the DAPT group.
Josep Rodés-Cabau MD et al. 2017 (14) (ARTE)	222	SAPT	Aspirin or acetylsalicylic acid (80–100 mg/day)	DAPT	Aspirin or acetylsalicylic acid (80–100 mg/day) plus clopidogrel (75 mg/day)	12	The primary endpoint was the rate of death, MI, ischemic stroke or TIA, or major or life threatening bleeding at 3-month follow-up.	Transfemoral: 72.1% in the DAPT group and 65.8% in the SAPT group. Via Transapical approach: 16.2% in the DAPT group and 18.0% in the SAPT group. Via Transaortic approach: 9.0% in the DAPT group and 12.6% in the SAPT group. Via Transcarotid approach: 2.7% in the DAPT group and 3.6% in the SAPT group.
Rogers Toby et al. 2021 (15)	94	SAPT	Aspirin 75–100 mg daily and 3–6 months of clopidogrel (75 mg daily)	VKA + SAPT	N/A	15	The primary effectiveness end point of the study was a composite of the following at 30 days: HALT, at least moderate RELM, hemodynamic dysfunction, stroke, or transient ischemic attack.	Transfemoral
Ole De Backer et al. 2020 (16) (GALILEO-4D)	231	SAPT	Aspirin at a dose of 75–100 mg daily and clopidogrel at a dose of 75 mg. once daily for 3 months. Followed by aspirin monotherapy (75–100 mg aily)	Rivaroxaban	Rivaroxaban at a dose of 10 mg aily plus aspirin at a dose of 75–100 mg daily or 3 months, followed by rivaroxaban monotherapy (10 mg daily)	3	At least 1 prosthetic valve leaflet with RELM of grade 3 or 4 or HALT of grade 3 or 4	N/A
Jorn Brouwer et al. 2020 (17) (Popular TAVI)	665	SAPT	Aspirin at a dose of 80–100 mg daily	DAPT	Aspirin–clopidogrel group were assigned to receive aspirin at a dose of 80–100 mg daily plus clopidogrel at a dose of 75 mg daily for 3 months, followed by aspirin alone (80–100 mg daily) for the entire duration of the trial	12	The two primary outcomes were all bleeding (including minor, major, and life-threatening or disabling bleeding) and non–procedure-related bleeding over a period of 12 months. The first secondary outcome was a composite of bleeding or thromboembolic events The other secondary outcome was a composite of death from cardiovascular causes, ischemic stroke, or myocardial infarction	N/A

TIA, transient ischemic attack; HALT, hypoattenuated leaflet thickening; RELM, reduced leaflet motion; SAPT, single antiplatelet therapy; DAPT, dual antiplatelet therapy (DAPT); OAC, oral anticoagulant; DOACs, direct oral anticoagulants; VKA, vitamin K antagonist; DVT/PE, deep vein thrombosis and pulmonary embolism.

**Table 2:** Baseline for inclusion in the study.

Characteristics	Duk Woo Park et al. (ADAPT-TAVR)		GeorgeD Dangas et al. (GALILEO)		Gian Paolo Ussia et al.		Josep Rodés Cabau et al. (ARTE)		Rogers T et al.		Becker et al. (GALILEO-4D)		Jorn Brouwer et al.	
	NOAC	DAPT	DOAC	SAPT	DAPT	SAPT	DAPT	SAPT	SAPT	OAC + SAPT	DOAC	SAPT	SAPT	DAPT
Age, (year)	80.2 (5.2)	80 (5.3)	80.4 (7)	80.8 (6)	81 (4)	80 (6)	79 (9)	79 (9)	73.1 (5.7)	73.6 (4.0)	79.7 (7.3)	80.5 (6.2)	80.4 (6.2)	79.5 (6.4)
Male, *n* (%)	49 (44.1)	47 (39.8)	426 (51.6)	405 (49.5)	16 (41)	20 (50)	70 (63.1)	59 (53.2)	37 (74.0)	29 (65.9)	74 (64.3)	74 (63.8)	167 (50.5)	174 (52.1)
BMI, (kg/m2)	24.(3.8）	24.8 (4.3)	28.1 (5.5)	28.2 (5.7)					30.1 (5.6)	32.0 (6.8)	27.7 (6.5)	27.8 (5.1)	27.0 (4.7)	27.1 (4.6)
Hypertension, *n* (%)	7 (6.3)	7 (5.9)	720 (87.2)	697 (85.2)	31 (80)	35 (88)	86 (77.5)	87 (79.8)	39 (78.0)	36 (81.8)	98 (85.2)	95 (81.9)	243 (73.4)	255 (76.3)
Diabetes, *n* (%)	81 (73.0)	84 (71.2)	236 (28.6)	235 (28.7)	8 (21)	13 (33)	41 (36.9)	36 (32.7)	15 (30.0)	15 (30.0)	21 (18.3)	27 (23.3)	78 (23.6)	85 (25.4)
Congestive heart failure, *n* (%)	35 (31.5)	36 (30.5)	394 (47.7)	380 (46.5)	14 (36)	18 (45)					52 (45.2)	52 (44.8)		
Ejection fraction (mean)	17 (15.3)	12 (10.2)	57.4 (10.9)	58.2 (11.2)	54 (8)	51 (12)	55 (12)	54 (13)	64.2 (8.9)	67.1 (3.1)	55 (11)	56 (10)		
NYHA III/IV, *n* (%)	64.4 (10.0)	64.2 (9.5)	250 (30.3)	222 (27.1)	23 (59.0)	26 (65.0)			12 (24.0)	8 (18.2)			212 (64.0)	220 (65.9)
Previous stroke, *n* (%)	30 (27.0)	31 (26.3)	51 (6.2)	35 (4.3)	4 (10.0)	2 (5.0)			0 (0.0)	1 (2.3)	11 (9.6)	6 (5.2)		
PVD, *n* (%)			83 (10.0)	82 (10.0)	4 (10.0)	3 (8.0)	28 (25.2)	22 (20.0)	1 (2.0)	1 (2.3)	10 (8.7)	10 (8.6)	47 (14.2)	68 (20.4)
VTE, *n* (%)	7 (6.3)	11 (9.3)	18 (2.2)	15 (1.8)	7 (18)	10 (25.0)					1 (0.9)	1 (0.9)		
COPD, *n* (%)			110 (13.3)	88 (10.8)			33 (30.0)	28 (25.2)			19 (16.5)	16 (13.8)	52 (15.7)	74 (22.2)
Mean GFR (ml/min/1.73 m^2^)			73.4 (23.8)	73.2 (23.2)							73.6 (19.2)	76,6 (19.4)		
CKD, *n* (%)					5 (13.0)	6 (15.0)			1 (2.0)	1 (2.3)				
STS core			4.0 (3.2)	4.3 (3.5)	8 (5.0)	7 (3.0)	6.2 (4.4)	6.4 (4.6)	1.5 (0.5)	1.4 (0.5)	2.8 (1.5)	3.0 (2.1)	2.6 (1.6)	2.4 (1.7)
PCI, *n* (%)	3.1 (2.1)	3.5 (2.7)			9 (23.0)	12 (30.0)			6 (12.0)	4 (9.1)				
Previous pacemaker, *n* (%)	18 (16.2)	14 (11.9)	80 (9.7)	80 (9.8)	1 (3.0)	4 (10.0)			1 (2.0)	0 (0.0)	14 (12.2)	14 (12.1)		
Euro SCORE	13 (11.7)	13 (11.0)	4.1 (3.9)	4.1 (3.7)	21 (16)	23 (15)								
Aortic valve area, (cm^2^)	2.3 (3.5)	2.4 (2.1)							0.85 (0.2)	0.82 (0.2)	1.8 (0.5)	1.8 (0.5)		
Mean gradient post procedure(mm Hg)	1.7 (0.4)	1.6 (0.4)	10.1 (4.7)	10.1 (4.6)			10.8 (5.3)	10.3 (5.7)						
Paravalvular regurgitation post, *n* (%)Mild			157 (19.0)	168 (20.5)			28 (29.2)	24 (25.0)			19 (16.5)	19 (16.4)		
Moderate or severe			10 (1.2)	10 (1.2)			9 (9.4)	6 (6.3)			2 (1.7)	1 (0.9)		
Previous CABG, *n* (%)	3 (2.8)	3 (2.7)			4 (10)	2 (5)	39 (35.1)	42 (38.5)					61 (18.4)	65 (19.5)
Previous myocardial infarction, *n* (%)	2 (1.8)	3 (2.5)			4 (10)	7 (18)	26 (23.4)	20 (18.4)	2 (4.0)	0 (0.0)			28 (8.5)	31 (9.3)
Previous TIA, *n* (%)	1 (0.9)	2 (1.7)			2 (5)	2 (5)								
Previous history of heart surgery, *n* (%)					1 (3)	2 (5)								

BMI, body mass index; PVD, peripheral vascular disease; VTE, venous thromboembolism; COPD, chronic obstructive pulmonary disease; GFR, glomerular filtration rate; CKD, chronic kidney disease; STS, society of thoracic surgeon; PCI, percutaneous coronary intervention; CABG, coronary artery bypass grafting; TIA, transient ischemic attack.

### Bias risk assessment

Because all of them are open labels, the overall implementation bias risk is high, and there are other bias risks in Dangas 2019, Josep 2017, and Rogers 2021. See Figure 2 for details.

### Safety endpoints

**Figure 3:**
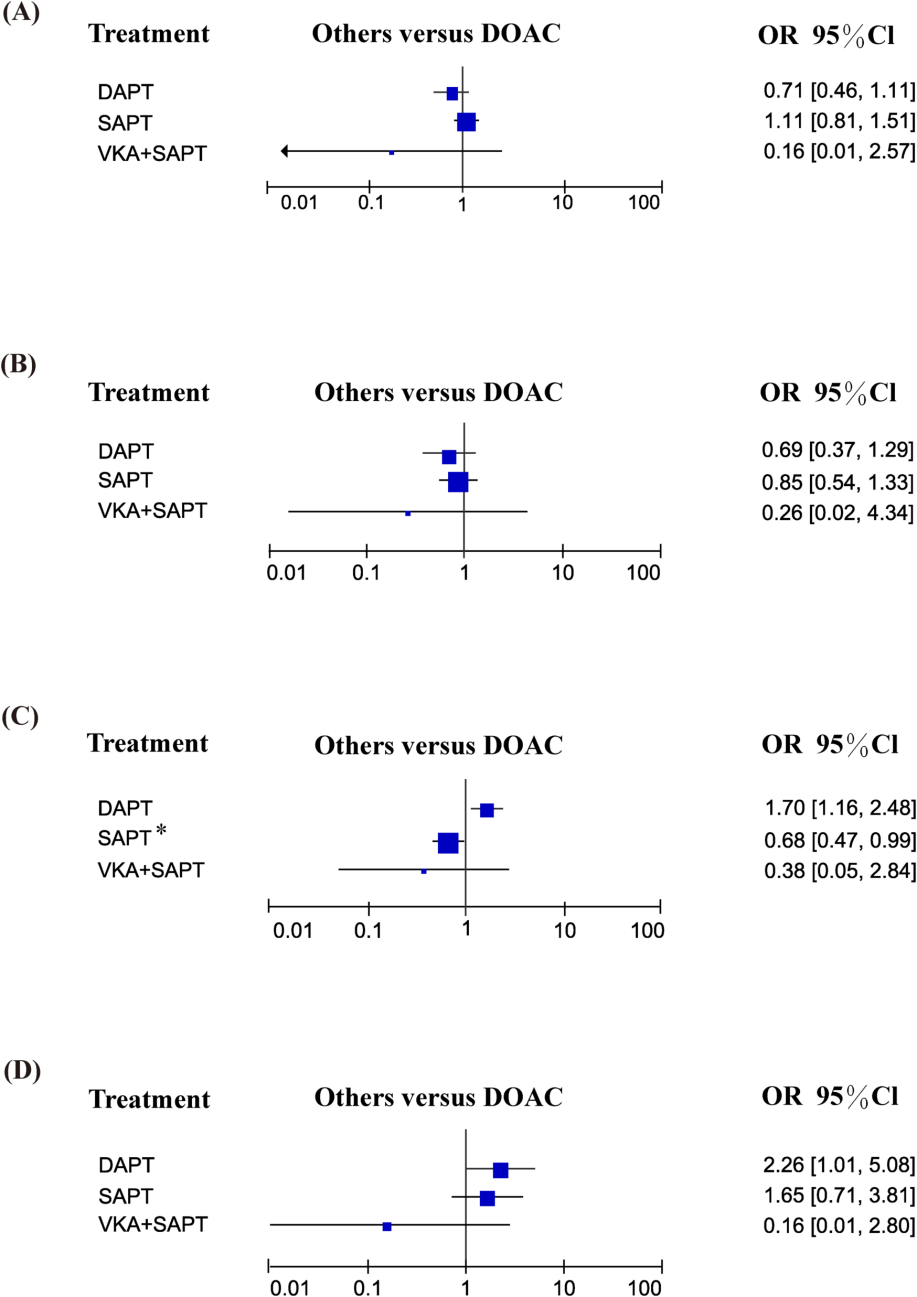
Safety endpoints: **(A)** all-cause mortality; **(B)** cardiovascular mortality; **(C)** major bleeding and life-threatening bleeding; **(D)** minor bleeding.

This study included all-cause mortality events in seven studies, cardiovascular death in four studies, major bleeding and life-threatening bleeding in seven studies, and minor bleeding in three studies. Compared with DOAC, there were no significant differences in all-cause mortality, cardiovascular mortality, and minor bleeding safety events. The rates of major bleeding and life-threatening bleeding in the SAPT group were lower than those in the DOAC group (OR: 0.68; 95% CI: 0.47–0.99). A detailed summary is provided in Figure 3.

### Efficacy endpoints

**Figure 4:**
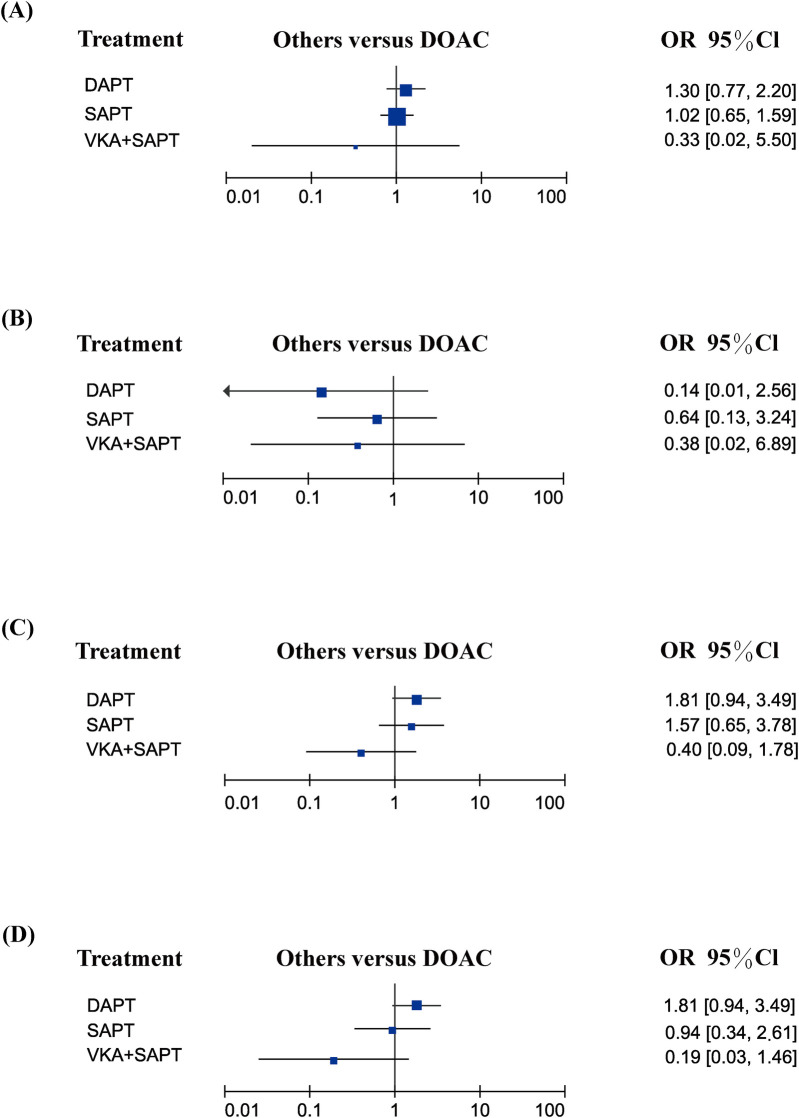
Efficacy endpoints: **(A)** ischemic stroke/TIA; **(B)** systemic embolism; **(C)** HALT; **(D)** RELM.

Among the efficacy endpoints, ischemic stroke/TIA was reported in seven studies, systemic embolism in two studies, HALT in three studies, and RELM in three studies. Compared to DOAC, no significant differences were observed in any of these endpoints (all *p* > 0.05). Comprehensive comparisons are detailed in Figure 4.

## Discussion

This network meta-analysis evaluated the safety and efficacy of various antithrombotic regimens for patients without an indication for OAC after TAVR. Seven randomized controlled trials were included in this analysis. The results indicated that patients receiving SAPT demonstrated lower incidence of major and life-threatening bleeding 3–24 months post-TAVR compared to those treated with DOAC therapy.

Currently, large-scale RCTs establish TAVR as the leading transcatheter approach for aortic valve replacement in symptomatic patients (18, 19). Despite its success in treating aortic stenosis, the optimal antithrombotic strategy to balance thrombosis and bleeding risks post-procedure remains uncertain (20, 21).

The basic principle of antithrombotic therapy after TAVR is to balance the prevention of leaflet thrombosis (driven by the thrombogenicity of bioprosthetic valve materials and altered hemodynamics) with the risk of bleeding (22). Currently, the ESC/EACTS 2021 and American College of Cardiology/American Heart Association (ACC/AHA) guidelines recommend SAPT for patients not on anticoagulation after TAVR (5). While previous studies suggest that DOACs may reduce bleeding events compared to VKAs (23), our analysis demonstrates that SAPT achieves an optimal balance between preventing thrombotic events and minimizing bleeding risks compared to DOACs, aligning with the primary goal of post-TAVR antithrombotic management. This finding is consistent with Rodes-Cabau et al.'s study (14), which compared SAPT with DAPT, further supporting guideline recommendations to prioritize SAPT in patients without OAC indications. Patient-specific factors, including advanced age and comorbidities (e.g., atrial fibrillation requiring OAC), as well as the use of non-DOAC anticoagulants (e.g., VKAs), are independently associated with elevated post-TAVR bleeding risk (24). Given that most TAVR recipients are elderly with multiple complications, tailoring treatment to individual anticoagulation needs is crucial to mitigate both bleeding and thromboembolic risks (6).

Subclinical leaflet thrombosis after TAVR is frequently observed (25). This condition causes HALT, visible via 4D computed tomography (CT) imaging, with or without RELM (26, 27). Observational studies report a correlation between stroke or TIA after TAVR and valvular thrombosis, with stroke or TIA increasing the incidence of valvular thrombosis (27). Our network meta-analysis found no significant difference in ischemic stroke or TIA events, systemic embolism, HALT, and RELM between DOAC and other treatment strategies. DOACs were not associated with a reduction in the incidence of RELM and HALT compared to other treatment strategies, which differs from the findings of Mohamed Abuelazm's meta-analysis (7). The research compared DOACs with standard care, including vitamin K antagonists, DAPT, or SAPT, but did not specifically compare SAPT, DAPT, and VKA groups. Our comparison of standard care details among groups may explain the different results. The impact of Subclinical leaflet thrombosis on clinical outcomes after TAVR remains unclear, with some reports suggesting it is not a decisive factor in evaluating different antithrombotic methods (26). In the future, subclinical leaflet thrombosis may be a valuable antithrombotic indication for post-TAVR patients. However, randomized controlled studies are needed to clarify the relationship between stroke or TIA and valvular thrombosis.

In summary, our network meta-analysis indicates that DOACs are less effective than SAPT regarding safety and efficacy in patients without anticoagulation indications after TAVR. The impact of subclinical leaflet thrombosis on clinical outcomes after TAVR remains unclear and requires further investigation.

## Limitations

Our research has several limitations. Firstly, as a network meta-analysis, we have limited ability to explain the heterogeneity among studies. There were some anticoagulated patients in Popular TAVI (subgroup A), which may increase the heterogeneity of our study. Secondly, we included seven randomized controlled trials but excluded retrospective studies. Additionally, the sample size was insufficient, limiting the generalizability of our results. Finally, the study varied in terms of timing, usage and dosage, valve type, and patient risk levels related to antithrombotic strategies. Future research is needed to further verify these results.

## Conclusion

The results of the network meta-analysis indicate that DOAC is less effective than SAPT for safety and efficacy in patients without anticoagulation indications following TAVR. Thus, SAPT remains the optimal choice for postoperative care.

## Data Availability

The original contributions presented in the study are included in the article/Supplementary Material, further inquiries can be directed to the corresponding author.
