# Supplementary material for: Antithrombotic strategies after transcatheter aortic valve replacement a network meta-analysis
Source: Front Cardiovasc Med. 2025 May 9;12:1496334. doi: 10.3389/fcvm.2025.1496334 (PMC12098576; doi:10.3389/fcvm.2025.1496334)
Supplement: Supplementary file 1 [file Table1.docx]

**Supplementary Table 1**

| Database | Search Terms | Search Results | | |
| --- | --- | --- | --- | --- |
| Pubmed | (Ticagrelor OR Clopidogrel OR Aspirin OR antiplatelet OR VKA OR coumadin OR warfarin OR DOAC OR Bevyxxa OR betrixaban OR Savaysa OR edoxaban OR Eliquis OR apixaban OR Xarelto OR rivaroxaban OR Pradaxa OR dabigatran OR Direct oral anticoagulants AND (TAVI OR transcatheter aortic valve replacement OR TAVR) |  | 37 |  |
| Cochrane | (Ticagrelor OR Clopidogrel OR Aspirin OR antiplatelet OR VKA OR coumadin OR warfarin OR DOAC OR Bevyxxa OR betrixaban OR Savaysa OR edoxaban OR Eliquis OR apixaban OR Xarelto OR rivaroxaban OR Pradaxa OR dabigatran OR Direct oral anticoagulants) AND (TAVI OR transcatheter aortic valve replacement OR TAVR) |  | 173 |  |

TAVR: transcatheter aortic valve replacement; TAVI: transcatheter aortic valve implantation; VKA: vitamin K antagonist
